# Multiple Episodes of Presyncope in a Pacemaker Dependent Patient: What is the Diagnosis?

**Published:** 2010-05-05

**Authors:** Siva K Mulpuru, Cesare Saponieri

**Affiliations:** 1 Fellow, Cardiovascular Diseases, Beth Israel Medical Center and Long Island College Hospital, Brooklyn, NY; 2 Attending, Cardiac Electrophysiology, Division of Cardiology, Long Island College Hospital, Brooklyn, NY

## Question

An 85 year old male presents to the emergency room with presyncopal episode associated with nausea and diaphoresis that lasted for 10 minutes. He has past medical history of hypertension and paroxysmal atrial fibrillation on anticoagulation. He also had complete AV block that necessitated the implantation of a dual chamber pacemaker (Medtronic Kappa KDR 701) 6 years prior to current presentation. The lead parameters were within normal limits during regular follow up every six months.  A recent echocardiogram suggested dilated left atrium and ventricle with preserved systolic function. His home medications included coumadin 5mg once daily and amlodipine 10mg once daily. The physical examination was unremarkable and orthostatic blood pressure measurements were within normal limits. All laboratory parameters were within normal limits. The electrocardiogram suggested atrial synchronous right ventricular apical pacing.

**Figure 1:**
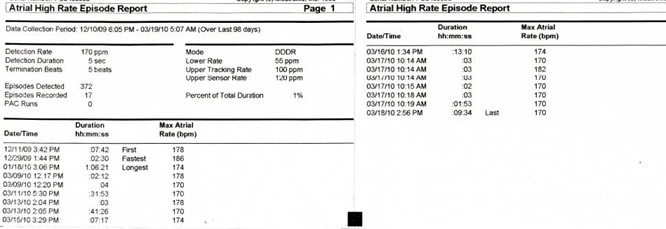
Arrhythmia burden over 3 months. The last episode corresponds to the time of symptoms

**Figure 2:**
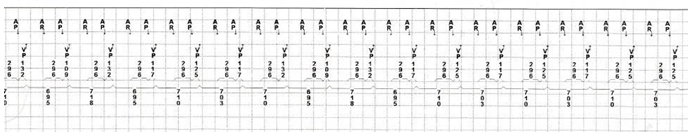
Intracardiac recording with marker channels during the arrhythmia episode.

On further questioning he has reported multiple presyncopal episodes in the last three months (Figure 1) that lasted about 10 minutes with no particular relationship to rest or exertion. A continuous rhythm strip that correlated to time of his symptoms is presented in Figure 2. The pacemaker was programmed to DDDR mode with lower rate of 55bpm, upper tracking rate of 100bpm and upper sensor rate of 120bpm. Mode switch rate was set at 175bpm. AV delay was fixed, programmed to 200msec. Sensed off set was programmed to 20msec.

Which of the following statements is correct regarding the patient's arrhythmia episode?

1. Patient has recurrent highly symptomatic atrial arrhythmias. Consider rhythm control strategy (drug versus catheter ablation)
2. Patient has pacemaker mediated tachycardia (PMT) and can be prevented by reducing the PVARP (post ventricular atrial refractory period).
3. Atrial lead is defective and consider lead revision.
4. The arrhythmia in the rhythm strip can be effectively treated by lowering basal rate, turning on rate adaptive short AV delay.
5. There is no evidence of pacemaker malfunction. Consider HUTT (Head up Tilt Table test) to evaluate for vaso-vagal syncope.

## Answer

The arrhythmia in the rhythm strip can be effectively treated by lowering basal rate, turning on rate adaptive short AV delay.

## Discussion

The electrogram recorded by the device at the time of symptoms suggests a distinct pattern in the atrial channel. Although there are two atrial events for every ventricular event, the pattern of long, short intervals between atrial events suggest a very specific form of endless loop tachycardia (ELT), termed RNRVAS (repetitive non reentrant ventriculo-atrial synchrony). It is also known as AV desynchronization arrhythmia or pseudo atrial exit block.

RNRVAS occurs due to functional atrial undersensing along with functional atrial non-capture as the atrial stimulus falls in the absolute refractory period of the myocardium. It is macrorentrant tachycardia using ventricular pacing as anterograde limb along with intact long intrinsic VA conduction as the retrograde limb. The retrograde atrial depolarization falls in the refractory period leading to functional undersensing. The circuit is completed when an atrial stimulus with functional non-capture triggers a ventricular stimulus at the end of programmed AV interval. Although upper rate pacing is not seen with this specific form of ELT, the hemodynamics are similar to pacemaker syndrome. The loss of AV synchrony leads to symptoms of dizziness and syncope. Activation of neurocardiogenic reflexes can cause nausea, vomiting and diaphoresis.

Even though the device classified the episode as high atrial rate event, presence of atrial pacing counters make it unlikely. In this situation, catheter ablation or antiarrhythmic medication would not improve symptoms. The reduction in PVARP leads to sensing of retrograde atrial event with ELT at the upper rate limit with similar hemodynamics. It is important to recognize RNRVAS and to not interpret it as device malfunction requiring atrial lead revision.

Shortening AV delay, reducing the lower rate to allow for atrial myocardium to recover following a retrograde sensed event, lowering upper sensor driven rate can help in prevention of recurrences. Algorithms to prevent RNRVAS include extension of PVARP following PVC (PVARP+), non competitive atrial pacing (NCAP- to allow for atrial myocardium to recover) and synchronous atrial pacing on detection of PVC (functional non capture of retrograde VA impulse). Rate adaptive AV delays, ELT termination algorithms may help if they allow atrial myocardium to recover from functional non capture.

